# Network pharmacology and experimental verification-based strategy to explore the underlying mechanism of Liu Jun An Wei formula in the treatment of gastrointestinal reactions caused by chemotherapy for colorectal cancer

**DOI:** 10.3389/fphar.2022.999115

**Published:** 2022-09-20

**Authors:** Gaobiao Li, Liying Liu, Yiran Yin, Mengmeng Wang, Lei Wang, Jianwei Dou, Hongwei Wu, Yufei Yang, Bin He

**Affiliations:** ^1^ Xiyuan Hospital, China Academy of Chinese Medical Sciences, Beijing, China; ^2^ Graduate School, Beijing University of Chinese Medicine, Beijing, China; ^3^ Dongzhimen Hospital, Beijing University of Chinese Medicine, Beijing, China; ^4^ School of Pharmacy, Xi’an Jiaotong University, Xi’an, Shaanxi, China; ^5^ Institute of Chinese Materia Medica, China Academy of Chinese Medical Sciences, Beijing, China

**Keywords:** Liu Jun An Wei formula (LJAW), network pharmacology, gastrointestinal reactions, colorectal cancer, target identification, intestinal organoid

## Abstract

**Background:** Liu Jun An Wei formula (LJAW), derived from “Liu Jun Zi Decoction”, is a classical prescription of Tradition Chinese Medicine and has been used for the treatment of gastrointestinal reactions caused by chemotherapy for colorectal cancer (CRC) for many years. Its molecular mechanism remains to be further explored.

**Objective:** To clarify the mechanism of LJAW in attenuating gastrointestinal reactions caused by chemotherapy for CRC.

**Methods:** The 5-fluorouracil (5-FU) induced mouse and intestine organoid models were established to observe the effect of LJAW. The ingredients of LJAW were analyzed and identified by UPLC-Q-TOF-MS technology. Targets of LJAW and chemotherapy-induced gastrointestinal reactions were collected from several databases. “Ingredient-target” network and protein-protein interaction network were constructed based on network pharmacology. Then, gene ontology (GO) functional analysis and Kyoto Encyclopedia of Genes and Genomes (KEGG) pathway analysis were performed. Subsequently, molecular docking method was used to verify the interaction between the core ingredients and key targets. The results were validated by both *in vivo* experiments and organoid experiments. Western Blot was used to analyze the influence of LJAW on key targets including PI3K, AKT1, MAPK1, MAPK14 proteins and their phosphorylated proteins. RT-qPCR and Western Blot were used to detect the mRNA and protein levels of apoptosis-related gene PUMA.

**Results:** Compared with the 5-FU group, the LJAW group had better morphology in mouse small intestine and intestine organoids. In total, 18 core ingredients and 19 key targets were obtained from 97 ingredients and 169 common targets. KEGG analysis showed that the common targets were involved in PI3K/Akt, MAPK, apoptosis and other signal pathways, which are closely related to gastrointestinal injury. Experiments confirmed that LJAW lowered the expressions of phosphorylated proteins including p-PI3K, p-AKT1, p-MAPK1, and p-MAPK14 and reduced the mRNA and protein levels of PUMA.

**Conclusion:** LJAW shows protective effect on 5-FU induced small intestine and intestinal organoids injury. LJAW attenuates gastrointestinal reactions caused by chemotherapy for CRC probably by regulating apoptosis-related genes through PI3K/AKT and MAPK signaling pathways.

## 1 Introduction

Colorectal cancer (CRC) is a malignant tumor with a high incidence rate in the digestive tract. According to the latest global cancer statistics report released by the International Agency for Research on *Cancer* (IARC) of the World Health Organization (WHO), CRC ranks third and second in the world’s cancer incidence and cause of death spectrum respectively, and ranks second and fifth in China respectively (Sung et al., 2021). CRC is considered a threatening disease that severely affects people’s health worldwide.

Chemotherapy plays an important role in the treatment of CRC. However, it often causes toxic side effects, such as gastrointestinal reactions (GR) and bone marrow suppression. The main GR caused by chemotherapy for CRC include chemotherapy-induced nausea and vomiting (CINV) and chemotherapy-induced diarrhea (CID). CINV is one of the most common adverse reactions in patients receiving chemotherapy. It is also a major concern for both patients and clinicians (Lorusso et al., 2017). The incidence rate of CINV ranges between 60% and 80% (Sommariva et al., 2016), and it directly affects the life quality of patients under treatment. These symptoms lead many patients to refuse to continue chemotherapy, which seriously affects their treatment (Inoue et al., 2015; Krikorian et al., 2019). The incidence rate of CID is as high as 50%–80% (Hoff et al., 2014), Among the patients with CID, about 60% have to change their chemotherapy regimen, 22% reduce their drug dose, even 15% stop the chemotherapy, and thus the prognosis of CRC patients is affected (Arbuckle et al., 2000; Escalante et al., 2017). 5-Hydroxytryptamine3 receptor antagonists like Ondansetron, NK-1 receptor antagonists like aprepitant, corticosteroids like dexamethasone, and dopamine receptor antagonists like metoclopramide are often used for CINV treatment (Shirley, 2021). The drugs used to alleviate CID include loperamide, octreotide, antibiotics, budesonide, and probiotics (Kordes and Gerling, 2019). However, they all have certain side effects, e.g., extrapyramidal symptoms, lengthening of the QT interval on the electrocardiogram, headache, and constipation. Currently, there is still no effective medication for refractory CINV and CID, resulting in a low degree of treatment satisfaction among relevant patients. Furthermore, there is no ideal drug for simultaneous CINV and CID.

A lot of Evidence-Based Medicine evidence have shown that Chinese herbal medicine may effectively attenuate GR caused by chemotherapy for CRC with high safety. This therapy improves the quality of life of patients and helps them to complete their chemotherapy smoothly without obvious side effects (Chen et al., 2018; Lin et al., 2019). Liu Jun An Wei formula (LJAW) is derived from a famous Tradition Chinese Medicine (TCM) prescription named “Liu Jun Zi Decoction”. In this new prescription, *Panax ginseng* C. A. Mey [Araliaceae; Ginseng Radix Et Rhizom] is changed into *Pseudostellaria heterophylla* (Miq.) Pax ex Pax et Hoffm [Caryophyllaceae; Pseudostellariae Radix, PR30 g] based on the TCM pathogenic characteristics during CRC chemotherapy. LJAW is composed of PR, *Atractylodes macrocephala* Koidz. [Compositae; Atractylodis Macrocephalae Rhizoma, AMR10 g], *Faria cocos* (Schw.) Wolf [Polyporaceae; Poria, PA10 g], *Pinellia ternate* (Thunb.) Breit [Araceae; Pinelliae Rhizoma Praeparatum Cum Zingibere Et Alumine, PRA10 g], *Citrus reticulata* Blanco [Rutaceae; Citri Reticulatae Pericarpium, CRP10 g], and *Glycyrrhiza uralensis* Fisch [Fabaceae; Glycyrrhizae Radix Et Rhizoma, GRER6 g]. According to the TCM theories, LJAW has the effects of invigorating the spleen, replenishing *Qi*, harmonizing the stomach, and lowering adverse *Qi*. It has been clinically used for the treatment of gastrointestinal symptoms caused by CRC chemotherapy, including nausea, vomiting, and diarrhea. In a previous study, our team found that LJAW exerted a protective effect on 5-fluorouracil (5-FU)-induced intestinal mucosal injury in mice (Zhao et al., 2021). Chinese botanical drugs are characterized by multi-target and multi-pathway action, and the mechanism of action for LJAW in the treatment of GR caused by chemotherapy for CRC is still not so clear. Therefore, it is of great significance to conduct an in-depth study on the mechanism of action for LJAW in such cases.

With the rapid development of biological information technology, network pharmacology has become an important means to study the mechanism of Chinese botanical drugs (Ma et al., 2022; Nogales et al., 2022). In the network pharmacology study, ultra high performance liquid chromatography-quadrupole-time of flight tandem mass spectrometry (UPLC-Q-TOF-MS) technology is adopted to analyze and identify the active ingredients of Chinese botanical drugs. Online databases are used to collect active pharmaceutical ingredients and related disease targets to systematically analyze Chinese botanical drugs through constructing relevant network with a visualization software. This methodology has been previously used to explore the mechanism of action for different drugs in the intervention of related diseases (Tong et al., 2021; Xia et al., 2021). In this study, UPLC-Q/TOF-MS was used to identify the main active ingredients of LJAW. Then the “ingredient-target” network and protein-protein interaction (PPI) network were constructed and the gene ontology (GO) and Kyoto Encyclopedia of Genes and Genomes (KEGG) analyses were conducted to predict the core pharmacodynamic ingredients, key targets, and related pathways of LJAW. Subsequently, the molecular docking method was used to verify the interaction between the core ingredients and key targets. At last, the molecular mechanisms of LJAW for the treatment of GR predicted by network pharmacology were verified through Western blot and real-time quantitative polymerase chain reaction (RT-qPCR) analyses based on *in vivo* and organoid experiments. The final results presented here provide basic research evidence for the clinical application of LJAW.

## 2 Materials and methods

### 2.1 Preparation of LJAW intestinal absorption liquid (LJAW-IAL)

A total of 380 g mixed prepared sliced crude botanical drugs consisting of PR (150 g), AMR (50 g), PA (50 g), PRA (50 g), CRP (50 g), and GRER (30 g) were extracted twice. They were mixed in 3,040 ml (eightfold greater than the total weight of the raw material) and then 1900 ml (fivefold greater than the total weight of the raw material) of 70% ethanol with refluxing, for 1 and 0.5 h, respectively. Each extraction was filtered using a 200-mesh filter and then combined. The ethanol solution was recovered by rotary evaporation under reduced pressure at 60°C. Then LJAW extract was obtained by vacuum drying apparatus under the same conditions. LJAW extract was dissolved in a Tyrode buffer (NaCl 8.00 g, KCl 0.28 g, NaHCO_3_ 1.00 g, NaH_2_PO_4_ 0.05 g, MgCl_2_ 0.10 g, dissolved in 500 ml water, sealed and refrigerated, CaCl_2_ 0.20 g, dissolved in 500 ml water, sealed and refrigerated, then the two solutions were mixed evenly before use (+glucose 1.00 g, fully dissolved, pH 7.2-7.4)) to prepare a test solution with a concentration of 1 g crude drug per milliliter solution for preparing LJAW-IAL.

The method for the preparation of LJAW-IAL was described in previous related studies (Zhang M. Y. et al., 2017). The rats were maintained in fasting conditions for 12 h before anesthesia. Then, the intestine of each rat was quickly removed and washed with Tyrode buffer. The intestines were cut into four 14 cm segments and made into sacs. The sacs were filled with Tyrode buffer and incubated in Magnus’ bath for 5 min to reach equilibration. The Tyrode buffer was replaced with the test solution of LJAW extract (25 ml), which was maintained at 37 °C and continuously injected with O_2_/CO_2_ (95%/5%). After 2 h, serosal side solutions containing the absorbed constituents were drained into tubes. Then, LJAW-IAL was filtered with a micro filtrate membrane (0.45 μm) and stored at -80°C.

### 2.2 Identification of the chemical components in LJAW-IAL by UPLC/Q-TOF-MS

#### 2.2.1 Chemicals and reagents

All organic solvents used in the experiments, including methanol, acetonitrile, and formic acid were LC/MS-grade and were purchased from Thermo Fisher Scientific Co., Ltd (Shanghai, China). The purified water was purchased from Wahaha Group Co., Ltd. (Hangzhou, China). All the standards used for Atractylenolide III, Hesperidin, Pentamethoxyflavone, and Liquiritin were all purchased from Chengdu Pusi Biotechnology Co., Ltd. (Chengdu, China), and the purity was greater than 98%. PR, AMR, PA, PRA, CRP, and GRER were obtained from the Chinese Pharmacy of Xiyuan Hospital, China Academy of Chinese Medical Sciences (Beijing, China).

### 2.2.2 Sample preparation for LC-MS analysis

Prior to the analysis, 100 μL LJAW-IAL and 300 μL of cold acetonitrile were mixed and vortexed for 30 s. The mixture was deproteinized by centrifugation at 4°C (21,130 g for 30 min), and the supernatant was put into a new centrifugal tube and dried using N_2_ at room temperature (25°C). The residue was then dissolved in 500 μl of methanol and injected into the LC-MS analysis system for the determination.

#### 2.2.3 Chromatographic and mass spectrometric conditions

Separation was performed by UPLC (U3000, Thermo Scientific, United States) and screened with ESI-MS. The LC system was made of a Waters ACQUITY BEH C18 Column (100 × 3 mm, 1.8 µm). The mobile phase was a mix of solvent A (0.1% formic acid-water) and solvent B (acetonitrile) with gradient elution (0–2 min, 5–5% B, 4–14 min, 20–25% B, 26–28 min, 46–100% B). At this stage, the flow rate of the mobile phase was 0.3 ml•min-1. The column temperature was maintained at 35°C, the sample manager temperature was set at 4°C, and the injection volume was 2 µl.

Mass spectrometry was performed on a Quadrupole/Time-Of-Flight Mass Spectrometer (Q/TOF-MS, model TripleTOF5600 + AB SCIEX™, United States) using a AB SCIEX ESI source. The instrument was operated in positive and negative-ion modes, respectively. The scanning mass-to-charge (m/z) range was from 50 to 1,500 with a scan rate of 1.00 spectra•sec-1. The capillary voltage was set to 5500 and 4400 V (positive and negative mode, respectively), TOF MS scan accumulation time 0.2 s, product ion scan accumulation time 0.01 s, Ion Source Gas1 (Gas 1):50 psi, Ion Source Gas2(Gas 2):50 psi, Curtain Gas (CUR): 25 psi, Source Temperature: 500 and 450°C (positive and negative mode, respectively).

Analyst 1.6 was used for data acquisition, Analyst 1.6 and Peak View 2.1. was used for mining. The components of LJAW mainly comprise the chemical substances from the single botanical drugs it contains. The chemical composition information of the herbal medicines involved in LJAW was summarized according to previous literature and related chemical databases (e.g., Scifinder, TCMID, CNKI, etc.) so the new compound database of LJAW could be established. The mass data of LJAW-IAL sample were aligned based on the m/z value and the retention time of the ion signals obtained with Peak View 2.1. Then, the chemical structures were identified according to the databases using the data of exact masses and the retention time. When necessary, further confirmation was acquired through comparisons with the authentic standards and MS/MS fragmentation patterns. Some tentatively identified compounds may have several possible isomers, which were counted as one compound. The measured masses were all within a mass deviation of 10 ppm, which made the characterization more reliable.

### 2.3 Prediction of ingredients and disease-related targets

For the active ingredients of LJAW-IAL, the corresponding CAS numbers were input into PubChem (https://pubchem. ncbi. nlm.nih.gov/). Then, the SMILES structures of the active ingredients of LJAW were downloaded from the platform and uploaded under “*Homo sapiens*” conditions to Swiss Target Prediction (http://www. swisstargetprediction. ch/). After this process, the targets related to the ingredients could be obtained and targets for each active ingredient with probability >0 were selected. CINV and CID were entered into the Drugbank (https://www. drugbank. ca/), GeneCards (https://www.genecards.org/), OMIM (http://omim.org), Pharmgkb (https:// www.pharmgkb.org/), and TTD databases (http://db.idrblab.net/ttd/) for search under “*Homo sapiens*” conditions. Last, these targets were normalized to the official gene symbol through the UniProt database (http://www.uniprot.org/). Common targets between ingredients and diseases were obtained by the construction of Venn diagrams.

### 2.4 Construction of the “ingredient-target” network

The Traditional Chinese Medicine Systems Pharmacology Database and Analysis Platform (http:/1spnwu.edu.cn/tcmspphp) were used for screening based on oral bioavailability >30% and drug likeness >0.18. An “ingredient-target” network was constructed using Cytoscape 3.7.2 software, with nodes representing ingredients or targets, and edges representing the connections between them. A statistical analysis was conducted based on network topology, with “*Degree (D)*”, “*Betweenness Centrality (BC)*” and “*Closeness Centrality (CC)*” used to assess the significance of ingredients. The core ingredients of LJAW for the treatment of GR caused by chemotherapy for CRC were screened out according to the analysis results.

### 2.5 Establishment of the PPI network

The common targets were uploaded to the STRING database (https://www.string-db.org/)) for the construction of a PPI network. The protein type was set to“ *Homo sapiens*”, the confidence level was set to “the highest confidence (0.900)”, and the isolated proteins in the network were hidden to obtain the PPI network. Relevant data from the network were input into Cytoscape3.7.2 software for network construction and topology analysis operation to screen out its key targets. The parameters for key target screening were the same as aforementioned - “D”, “BC” and “CC”. Regarding the screening conditions, the online program package of R software was used to work out D, BC and CC values for 169 intersection genes, and the genes with values greater than the median value of the group were reserved. The R script was run twice to screen out the key targets.

### 2.6 GO and KEGG enrichment analyses

The David6.8 database (https://david.ncifcrf.gov/) was used for GO functional enrichment and KEGG pathway enrichment. The GO analysis includes biological process (BP), cellular ingredient (CC), and molecular function (MF). The Clusters Profiler R software package was used to perform GO and KEGG analyses for the common targets. Relevant statistical significance threshold was set as *p* < 0.05.

### 2.7 Molecular docking verification

The 3D structures of the key targets were downloaded from PDB (http://www.rcsb.org/), and the core ingredients were processed into MOL2 files using Chembio Office software. Subsequently, hydrogenation, dehydration, and ligand separation were performed. Autodock Vina 1.1.2 software was used to conduct molecular docking between the key targets and the core ingredients and obtain their binding energy. Low negative binding energy was conducive to the binding of a compound to the target as well as conformation stability. The conformation with the best affinity was selected as the final docking conformation for visualization using PyMOL2.3.

### 2.8 Validation by *in vivo* experiment and intestinal organoids experiment

#### 2.8.1 Mice and drug intervention

In this study, we adopted male SPF C57BL/6J mice, age 7–8 weeks, body weight 18–20 g, which were purchased from SPF (Beijing) Biotechnology Co., Ltd., License number: SCXK (Beijing) 2019-0010. Environment for animal feeding: SPF laboratory animal room, room temperature: 24°C, relative humidity: (60 ± 5) %, 12 h/12 h light/dark cycle. The animals were free to eat and drink as needed. All the animal-based experiments were approved by the Ethics Committee of Xiyuan Hospital, China Academy of Chinese Medical Sciences and performed following the relevant guidelines and regulations. Our team took appropriate measures for alleviating pain levels.

The male C57BL/6J mice were fed adaptively for 1 week and then randomly divided into normal control group (NC), model group (5-FU), LJAW high-dose group (LJAW-H), LJAW medium-dose group (LJAW-M), and LJAW low-dose group (LJAW-L), with 10 mice in each group. The 5-FU, LJAW-H, LJAW-M, and LJAW-L needed 5-FU intervention, and the procedure for 5-FU modeling was conducted according to the following steps: Consecutive administration of intraperitoneal 5-FU injection for 7 days (day 8 to day 14), 50 mg/kg, once a day (Sangild et al., 2018). The successful modeling was verified by decreased body weight and diarrhea in mice in combination with microscopic pathological observation of their intestinal tissue. NC mice were injected with the same amount of the vehicle (normal saline) intraperitoneally for seven consecutive days, once a day.

Based on a human-mouse equivalent dose conversion, the dosage of LJAW was 15.66 g/kg, 7.83 g/kg, and 3.92 g/kg for LJAW-H, LJAW-M, and LJAW-L by gavage, once a day. The NC and 5-FU groups were given equal amounts of the vehicle (sterile water) by gavage for 14 consecutive days (day 1 to day 14). On Day 15, the mice were sacrificed by the cervical dislocation method for collecting their small intestines, which were fixed with 4% paraformaldehyde solution.

#### 2.8.2 Hematoxylin-eosin (HE) staining

After dissecting the mice, the jejunum tissue was taken and placed in 4% formaldehyde solution for 24 h. After fixation, the jejunum was dehydrated with gradient alcohol, embedded in paraffin, sectioned, and stained with conventional HE to observe the jejunal mucosal structure under a microscope at 50 magnifications. The villus and crypt structures were found at 400 magnifications, and the villus height and crypt depth were measured with Image-Pro P1us6.0 software.

#### 2.8.3 Organoid culture and model establishment

Small intestinal crypts of C57BL/6 mice were isolated *in vitro*, and intestinal organoids were obtained after 3–4 days of culture. These intestinal organoids were then divided into normal control group (NC), model group (5-FU), LJAW treatment group (LJAW), and glutamine group (Gln) as positive control group. The NC group was cultured in the normal medium and the 5-FU group was treated with 1.0ug/mL of 5-FU for intervention (Mikawa et al., 2019). In the LJAW group, LJAW-IAL was added into the culture medium 24 h before adding 5-FU to make the final concentration within 0–40 mg/ml. The intervention was performed for 24 h, 48 h and 72 h. After intervention, the Methyl thiazolyl diphenyl-tetrazolium bromide (MTT) method was used to detect the OD values under various concentrations after different intervention hours, so it was possible to calculate the cell death rate in organoids. The Gln group was cultured with a medium containing 1.0 ug/mL of 5-FU and 12 mM of glutamine.

#### 2.8.4 MTT

The MTT solution was added to the organoid culture system at a final concentration of 500ug/ml. The system was incubated at 37°C and 5% CO_2_ for 1 h. After the MTT culture solution was completely removed, 80 μl of 2% SDS solution was added to each well, and incubated at 37°C and 5% CO_2_ for 1 h. To promote the dissolution of the matrix gel, the culture plates were gently shaken intermittently. Then, 320 μl of DMSO solution was added to each well, and incubated in an incubator for 1 h to dissolve the metabolite formazan. The absorbance values of wells in the NC group, the experimental group and those containing only matrix gel and culture medium were measured at 562 nm by a microplate reader, being labeled as OD (control), OD (sample), and OD (background, BG), respectively. The cell viability in organoids was calculated as per the following formula: (1-[OD (sample)-OD (BG)/(OD (control) - OD (BG)])*100%.

#### 2.8.5 Western Blot

Proteins were extracted from the mice’s small intestines and organoids according to the instructions of RIPA lysis buffer (Shanghai Beyotime Biotechnology Co., Ltd., Shanghai, China) and quantified using a BCA protein assay kit (Thermo Fisher Scientific, Shanghai, China). The proteins were isolated by SDS-PAGE (40 μg/lane) and transferred to the PVDF membrane, and then sealed with 5% skim milk in TBST buffer. After being kept at room temperature for 2 h, and the membrane was incubated with primary antibodies specific to proteins: PI3K, p-PI3K, AKT1, p-AKT1, MAPK1, p-MAPK1, MAPK14, p-MAPK14, and PUMA at 4°C overnight and washed with TBST for 3 times, 10 min each time. After that, the second antibodies were added, and the membrane was incubated for another hour, then washed with TBST for 3 times, 10 min each time. The enhanced chemiluminescence system and Image Pro-Plus were used for the imaging and quantification of protein bands, respectively.

#### 2.8.6 RT-qPCR

The total RNA was extracted with Trizol solution according to the instructions for use (Promega, Beijing, China), and the cDNA was synthesized using the first strand cDNA synthesis kit. qRT-PCR was performed using SYBR Green qPCR SuperMix. The reaction conditions were as follows: 50°C for 2 min, 95°C for 2 min, 95°C for 15 s, 60°C for 32 s, 40 cycles. The melting curves were analyzed at 60–95°C. Each sample was detected for three times by qRT-PCR (Biorad IQ5). The relative mRNA expression was normalized to the corresponding β-actin expression and analyzed by 2^−△△Ct^ method. The nucleotide sequences of primer pairs used to quantify gene expression were as follows:PUMA-F:5′-GGGTGTCTGCGTGAGTCC-3′, PUMA-R:5′-CCAGGCAAGCG ACAGA TAC A-3′, β-actin-F:5′-CTCTGTGTGGATCGGTGGCT-3′, β-actin-R:5′-TGTAAAACGCAGCTCAGTAA CAG-3'.

### 2.9 Statistical analysis

All data in the experiments were expressed as mean ± standard deviation. The one-way ANOVA with Tukey test was adopted for comparing multiple sets of independent data. All data were analyzed statistically with GraPhPad Prism 9.0 (GraPhPad software, San Diego, United States). For all comparisons, statistical significance was considered if *p* < 0.05.

## 3 Results

### 3.1 Protective effect of LJAW on small intestine injured by 5-FU

**FIGURE 1:**
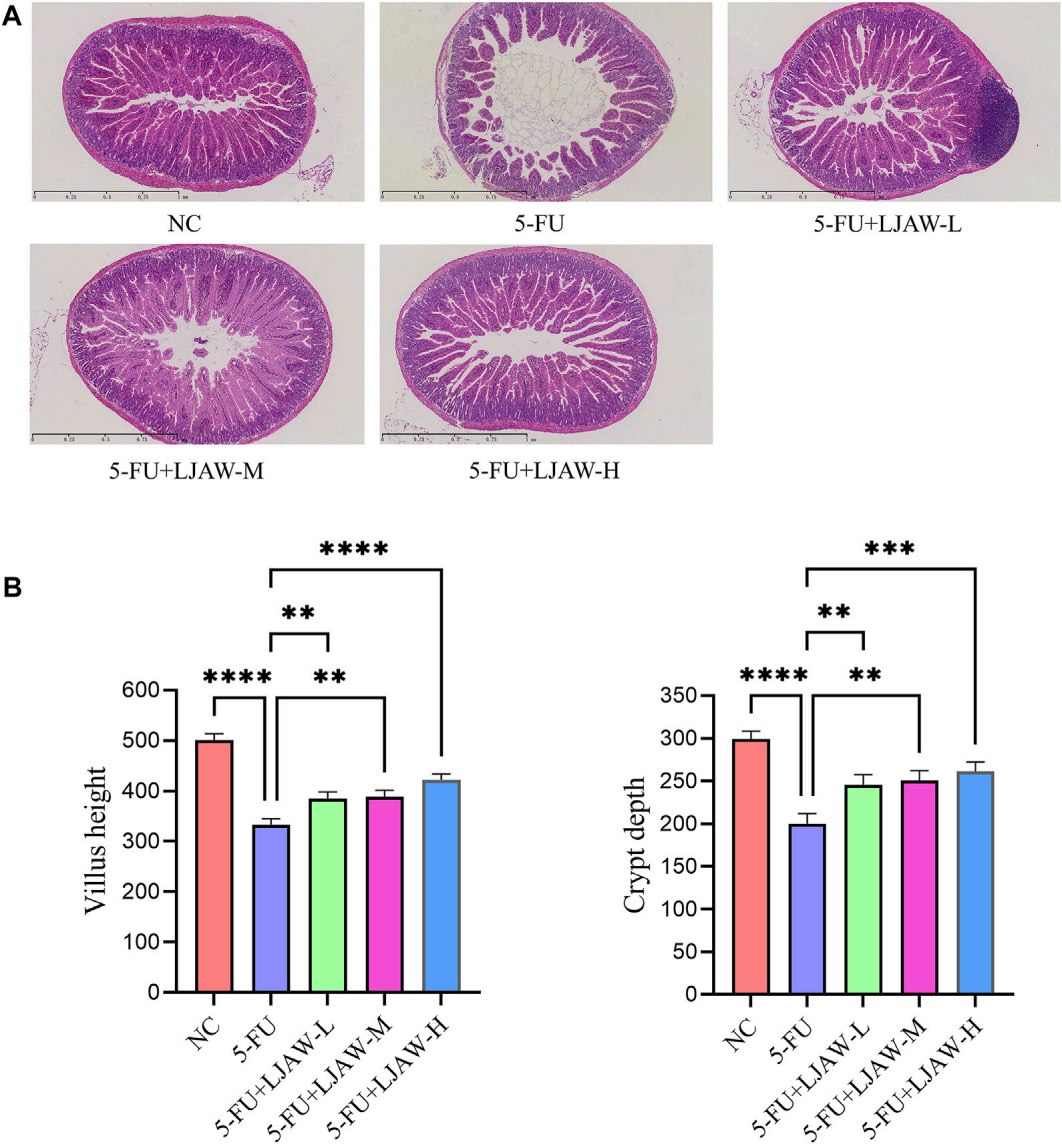
Protective effect of LJAW on 5-FU-induced small intestine injury **(A)** Effect of different doses of LJAW on the morphology of small intestinal mucosa in mice. NC: the normal control group, 5-FU: the model group, 5-FU + LJAW-H: the 5-FU + LJAW high-dose group, 5-FU + LJAW-M: the 5-FU + LJAW medium-dose group, 5-FU + LJAW-L: the 5-FU + LJAW low-dose group. **(B)** Comparison of villus height and crypt depth in different groups. Data are expressed as means ± SD (n = 3). **p < 0.01, ***p < 0.001 and ****p < 0.0001 compared with the 5-FU group.

The HE staining results showed that compared with the NC group, the 5-FU group had villus height shortened or atrophic villi (*p* < 0.05), wider villus spacing, crypt structure damaged or shallowed (*p* < 0.05), inflammatory cell infiltration in the submucosa, and mild disorder of smooth muscle arrangement in the muscle layer. Moreover, compared with the 5-FU group, the villus height of the LJAW group was higher (*p* < 0.05), the LJAW group had small villus spacing, mild crypt damage, and deep crypt (*p* < 0.05), submucosa infiltrated by a small number of inflammatory cells, and a regular muscle layer, the high dose group had the best efficacy (Figures 1A,B).

### 3.2 Protective effect of LJAW on intestinal organoids injured by 5-FU

**FIGURE 2:**
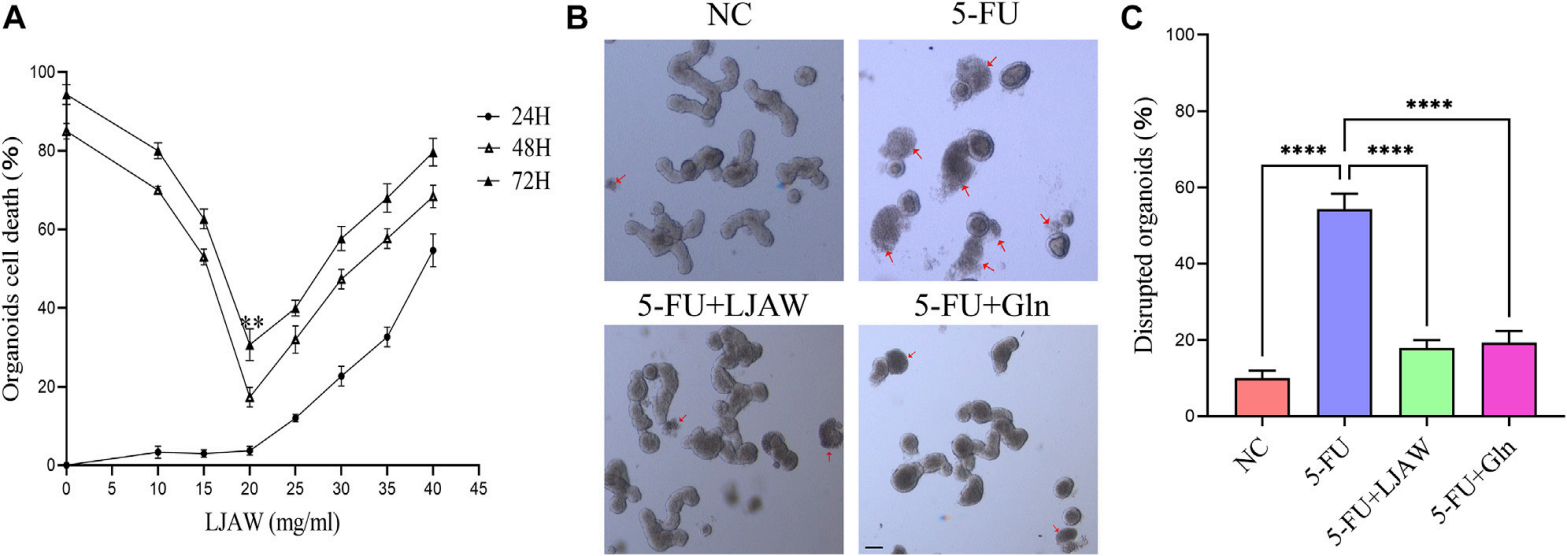
Protective effect of LJAW on 5-FU-induced intestinal organoids injury **(A)** Organoid cell death under different concentrations of LJAW after different intervention times detected by MTT, 24H: LJAW intervention for 24 h before adding 5-FU, 48H: LJAW intervention for 48 h, 5-FU intervention for 24 h, 72H: LJAW intervention for 72 h, 5-FU intervention for 48 h **(B)** and **(C)** Effect of LJAW on morphological of organoids. Count the total organoids and the number of disrupted organoids per well (the disrupted organoids are marked by red arrows). Scale bar, 100 μm. Data are expressed as means ± SD (n = 3). **p < 0.01 compared with the 48H group. ****p < 0.0001 compared with the 5-FU group.

The MTT results showed that after 24 h of LJAW intervention (without 5-FU), no significant cytotoxicity was identified in organoids from the LJAW group concentration range of 5–20 mg/mL as compared with the NC group. Furthermore, during the 48 and 72 h of LJAW (at this time, with 5-FU intervention for 24 and 48 h respectively) the cell death rate in organoids changed in a time-dependent manner. The lowest cell death rate in organoids happened in 48 h of 20 mg/ml LJAW intervention, indication that LJAW had the best protective effect at this specific concentration. Therefore, 20 mg/ml LJAW was selected for 48 h intervention as the subsequent intervention condition (Figure 2A). The organoids in the NC group grew well, exhibiting a good Crypt-Villus structure, similar to the first intestinal organoid cultured successfully (Sato et al., 2009). Compared with the NC group, the 5-FU group had severe morphological destruction of organoids, and more disrupted ones. In contrast, the LJAW group and Gln group had better morphology, and fewer disrupted ones (Figures 2B,C).

### 3.3 Identification of the chemical ingredients in LJAW-IAL by UPLC/Q-TOF-MS

**FIGURE 3:**
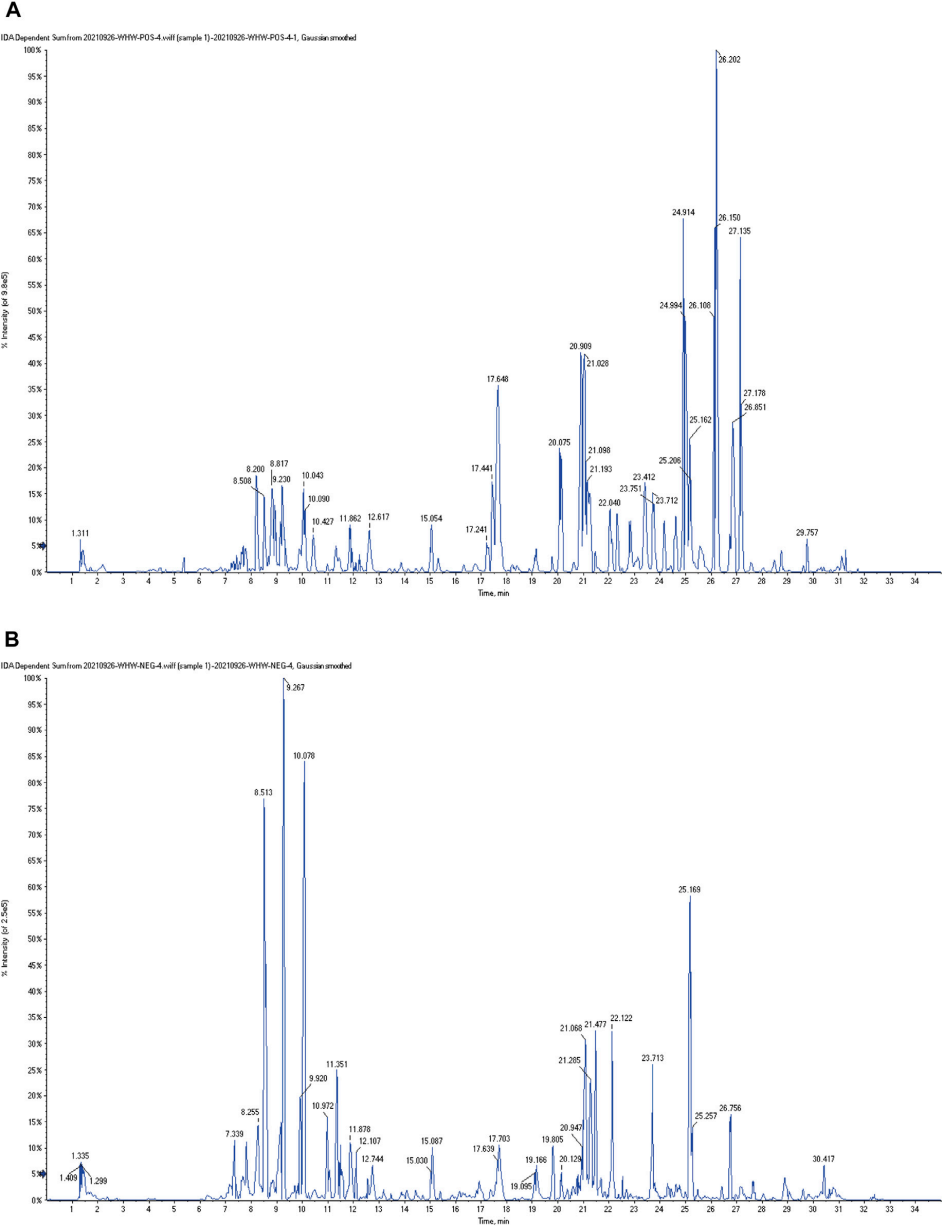
The typical total ion chromatography (TIC) profiles of LJAW-IAL sample **(A)** TIC of LJAW-IAL in positive ion mode. **(B)** TIC of LJAW-IAL in negative ion mode.

The typical total ion chromatography (TIC) profiles of LJAW-IAL sample in the positive and negative signal mode are illustrated in Figures 3A,B.

In the LJAW-IAL sample, a total of 97 compounds were preliminarily identified in LJAW-IAL, including 7 in PR, 6 in AMR, 5 in PA, 24 in PRA, 17 in CRP, and 50 in GRER. These mainly cover 48 kinds of flavonoids, 17 kinds of terpenes, 6 kinds of volatile oils, 6 kinds of gingerols, 6 kinds of amino acids and peptides, 7 kinds of carbohydrates, three kinds of phenylpropanoids, 2 kinds of coumarins, two kinds of alkaloids, 1 kind of sterols, and 16 other kinds. The names, structures, and additional detailed information of the identified compounds are shown in Table 1.

**TABLE 1:** 97 compounds in LJAW-IAL based on UPLC-Q-TOF MS.

Num.	Compound Name	Rt(min)	Formula	Ion Species	TheoreticalValue(m/z)	Measured value(m/z)	ppm	Source(ChineseMedicine)
1	L(+)-Arginine	1.29	C6H14N4O2	[M+H]^+^	175.11895	175.11812	-4.7	PR、PRA、AMR
2	Nystose	1.29	C24H42O21	[M+Na]^+^	689.21108	689.20545	-8.2	AMR
3	D-galactose	1.36	C6H12O6	[M+Na]^+^	203.05261	203.05133	-6.3	PRA
4	Alpha-sophorose	1.39	C12H22O11	[M+Na]^+^	365.10543	365.10377	-4.6	PR
5	Phenylalanine	4.06	C9H11NO2	[M+Na]^+^	188.0682	188.06943	6.6	PRA
6	Vicenin-2	7.44	C27H30O15	[M+H]^+^	595.16575	595.16232	-5.8	CRP、GRER
7	Cyclo-pro-val	7.56	C10H16N2O2	[M+HCOO]^-^	241.11828	241.11888	2.5	PRA
8	Narcissin	7.58	C28H32O16	[M-H]^-^	623.16176	623.16528	5.6	GRER
9	Schaftoside	7.81	C26H28O14	[M-H]^-^	563.14063	563.14356	5.2	GRER
10	Phenprobamate	7.92	C10H13NO2	[M+H]^+^	180.10191	180.1009	-5.6	PR、PRA、AMR
11	3-(4-hydroxy-3-methoxyphenyl)-propionic acid	8.17	C10H12O4	[M-H]^-^	195.06628	195.06634	0.3	PR、PRA
12	(E)-p-coumaryl alcohol	8.17	C9H10O2	[M+HCOO]^-^	195.06519	195.0663	5.7	PRA
13	Isoviolanthin	8.21	C27H30O14	[M-H]^-^	577.15628	577.15933	5.3	GRER
14	Rutin	8.28	C27H30O16	[M-H]^-^	609.14611	609.14999	6.4	PR、GRER
15	Scopoletin	8.28	C10H8O4	[M+H]^+^	193.04954	193.04833	-6.2	AMR、GRER
16	Ferulic acid	8.36	C10H10O4	[M-H]^-^	193.05063	193.05115	2.7	CRP、PRA
17	Eriocitrin	8.4	C27H32O15	[M-H]^-^	595.16684	595.17028	5.8	CRP
18	Liguiritigenin-7-O-D-apiosyl-4’-O-D-glucoside	8.48	C26H30O13	[M-H]^-^	549.16137	549.16425	5.2	GRER
19	Isoliquiritin apioside	8.48	C26H30O13	[M-H]^-^	549.16137	549.16425	5.2	GRER
20	Isoliquiritigenin	8.51	C15H12O4	[M+H]^+^	257.08084	257.07949	-5.3	GRER
21	Liquiritigenin	8.51	C15H12O4	[M+H]^+^	257.08084	257.07949	-5.3	GRER
22	Pentamethoxyflavone	8.55	C20H20O7	[M+HCOO]^-^	417.11801	417.12008	5	CRP
23	Liquiritin	8.55	C21H22O9	[M-H]^-^	417.11911	417.12008	2.3	GRER
24	Isoliquiritin	8.55	C21H22O9	[M-H]^-^	417.11911	417.12008	2.3	GRER
25	Neoliquiritin	8.55	C21H22O9	[M-H]^-^	417.11911	417.12008	2.3	GRER
26	Neoisoliquiritin	8.55	C21H22O9	[M-H]^-^	417.11911	417.12008	2.3	GRER
27	Neoisoliquiritin;isoneoliquiritin	8.55	C21H22O9	[M-H]^-^	417.11911	417.12008	2.3	GRER
28	Chrysoeriol-8-C-glucoside	8.79	C22H22O11	[M-H]^-^	461.10894	461.10968	1.6	CRP
29	Sophoraflavone B	8.79	C21H20O9	[M+HCOO]^-^	461.10784	461.10968	4	GRER
30	Diosmin	9.06	C28H32O15	[M+HCOO]^-^	653.17123	653.17481	5.5	CRP
31	Naringenin	9.26	C15H13O5	M^+^	273.07575	273.07362	-7.8	CRP、GRER
32	Naringin	9.27	C27H32O14	[M-H]^-^	579.17193	579.17486	5.1	CRP
33	Acaciin	9.67	C28H32O14	[M-H]^-^	591.17193	591.17435	4.1	PR
34	Wistin	9.83	C23H24O10	[M-H]^-^	459.12967	459.13125	3.4	GRER
35	5-Demethylnobiletin	9.96	C20H20O8	[M+HCOO]^-^	433.11292	433.11507	4.9	CRP
36	Choerospondin	9.96	C21H22O10	[M-H]^-^	433.11402	433.11507	2.4	GRER
37	Engeletin	9.96	C21H22O10	[M-H]^-^	433.11402	433.11507	2.4	PA
38	Hesperetin	10.05	C16H14O6	[M+H]^+^	303.08631	303.0849	-4.7	CRP
39	Hesperidin	10.07	C28H34O15	[M-H]^-^	609.18249	609.18512	4.3	CRP
40	Seselin	10.48	C14H12O3	[M+HCOO]^-^	273.07575	273.07767	7	PRA
41	Meranzin	11.2	C15H16O4	[M+H]^+^	261.11214	261.11009	-7.9	CRP
42	Chrysophanol	11.8	C15H10O4	[M-H]^-^	253.05063	253.05133	2.7	PRA
43	Formononetin-7-O-β-D-glucoside	11.86	C22H22O9	[M+H]^+^	431.13366	431.13007	-8.3	GRER
44	Licochalcone B	11.87	C16H14O5	[M-H]^-^	285.07685	285.07722	1.3	GRER
45	8-Methylretusin	12.21	C17H14O5	[M+H]^+^	299.0914	299.08859	-9.4	GRER
46	Osthol	12.36	C15H16O3	[M+HCOO]^-^	289.10705	289.10779	2.6	PRA
47	Licorice-glycoside B	12.54	C35H36O15	[M-H]^-^	695.19814	695.2014	4.7	GRER
48	Calycosin	13.39	C16H12O5	[M+H]^+^	285.07575	285.07375	-7	GRER
49	Poncirin	15.08	C28H34O14	[M-H]^-^	593.18758	593.1901	4.2	CRP
50	Galangin	16.29	C15H10O5	[M-H]^-^	269.04555	269.04575	0.7	GRER
51	Retrochalcone	16.97	C16H14O4	[M-H]^-^	269.08193	269.08231	1.4	GRER
52	Citrusin III	17.67	C36H53N7O9	[M-H]^-^	726.3832	726.38518	2.7	CRP
53	Methyl octanoate	18.75	C9H18O2	[M+Na]^+^	181.1199	181.12073	4.6	PRA
54	Atractylenolide III	19.07	C15H20O3	[M+H]^+^	249.14852	249.1465	-8.1	AMR
55	24-Hydroxy-licoricesaponin A3	19.1	C48H72O22	[M-H]^-^	999.44425	999.4514	7.2	GRER
56	Uralsaponin F	19.18	C44H64O19	[M-H]^-^	895.3969	895.40426	8.2	GRER
57	Licorice glycoside A	20.08	C36H38O16	M^+^	726.21544	726.22069	7.2	GRER
58	Melitidin	20.12	C33H40O18	[M-H]^-^	723.21419	723.21791	5.1	CRP
59	1-Isopropyl-2-methoxy-4- methyl-benzene	20.57	C11H16O	[M+HCOO]^-^	209.11722	209.11807	4.1	CRP
60	Heterophyllin B	21	C40H58N8O8	[M-H]^-^	777.43049	777.43431	4.9	PR
61	Licoricesaponin A3	21.07	C48H72O21	[M-H]^-^	983.44933	983.45607	6.9	GRER
62	Isoformononetin	21.27	C16H12O4	[M+H]^+^	269.08084	269.07977	-4	GRER
63	Columbianetin	21.27	C14H14O4	[M+Na]^+^	269.07843	269.07838	-0.2	PRA
64	Isovestitol	21.88	C16H16O4	[M-H]^-^	271.09758	271.09817	2.2	GRER
65	Citrusin I	22.05	C34H53N7O9	[M-H]-	702.3832	702.38803	6.9	CRP
66	Pinellic acid	22.45	C18H34O5	[M-H]^-^	329.23335	329.23404	2.1	PRA
67	Ergosterol-5,8-peroxide	23.59	C28H44O3	[M+Na]^+^	451.31827	451.31622	-4.5	PA
68	Licorice-saponin E2	23.63	C42H60O16	[M-H]^-^	819.38086	819.38706	7.6	GRER
69	Licoricesaponin G2	23.7	C42H62O17	[M-H]^-^	837.39142	837.39751	7.3	GRER
70	4-Gingerol	24.24	C15H22O4	[M-H]^-^	265.14453	265.14476	0.8	PRA
71	16 Alpha-ydroxytrametenolic Acid	24.28	C30H48O4	[M+Na]^+^	495.34448	495.33975	-9.6	PA
72	Glabridin	24.38	C20H20O4	[M+HCOO]^-^	369.13327	369.13474	4	GRER
73	Glyasperin B	24.38	C21H22O6	[M-H]^-^	369.13436	369.13474	1	GRER
74	Uralsaponin C	25.18	C42H64O16	[M-H]^-^	823.41216	823.4076	-5.5	GRER
75	Licorice saponin B2	26.41	C42H64O15	[M-H]^-^	807.41725	807.42129	5	GRER
76	Sigmoidin B	27.16	C20H20O6	[M-H]^-^	355.11871	355.11899	0.8	GRER
77	Licochalcone D	27.41	C21H22O5	[M+H]^+^	355.154	355.15049	-9.9	GRER
78	Pinoresinol	27.43	C20H22O6	[M-H]^-^	357.13436	357.13455	0.5	PRA
79	Glycycoumarin	27.63	C21H20O6	[M-H]^-^	367.11871	367.11988	3.2	GRER
80	Glabrene	27.63	C20H18O4	[M+HCOO]^-^	367.11762	367.11988	6.2	GRER
81	Gingerenone A	28.03	C21H24O5	[M-H]^-^	355.1551	355.15586	2.1	PRA
82	Licoisoflavone A	28.88	C20H18O6	[M-H]^-^	353.10306	353.10431	3.5	GRER
83	Licoflavonol	28.88	C20H18O6	[M-H]^-^	353.10306	353.10431	3.5	GRER
84	6-Gingesulfonic acid	28.88	C17H22O6S	[M-H]^-^	353.10643	353.10465	-5	PRA
85	Glycyrin	29.59	C22H22O6	[M-H]^-^	381.13436	381.13548	2.9	GRER
86	6,7-Dehydroporicoic Acid H	29.6	C31H45O5	M^-^	497.32725	497.33014	5.8	PA
87	Poricoic acid A	29.6	C31H46O5	[M-H]^-^	497.32725	497.33014	5.8	PA
88	Neoglycyrol	29.8	C21H18O6	[M-H]^-^	365.10306	365.10426	3.3	GRER
89	2-Cyclopentene-1-undecanoic acid,methyl ester	30.25	C17H30O2	[M+HCOO]^-^	311.22169	311.22289	3.9	PRA
90	Neryl propionate	30.3	C13H22O2	[M+Na]^+^	233.1512	233.15115	-0.2	PRA
91	Licoisoflavone B	30.43	C20H16O6	[M-H]^-^	351.08741	351.08801	1.7	GRER
92	Semilicoisoflavone B	30.43	C20H16O6	[M-H]^-^	351.08741	351.08801	1.7	GRER
93	Glabrone	30.63	C20H16O5	[M-H]^-^	335.0925	335.09299	1.5	GRER
94	Hexahydrocurcumin	30.81	C23H30O6	[M+Na]^+^	425.19346	425.19538	4.5	PRA
95	GancaoninE	30.82	C25H28O6	[M-H]^-^	423.18131	423.18388	6.1	GRER
96	Glycol monopalmitate	31.42	C18H36O3	[M+Na]^+^	323.25567	323.25367	-6.2	PRA
97	Spathulenol	32.93	C15H24O	[M-H]^-^	219.17544	219.17563	0.9	AMR、PRA

### 3.4 Common targets of LJAW and GR caused by chemotherapy for CRC

**FIGURE 4:**
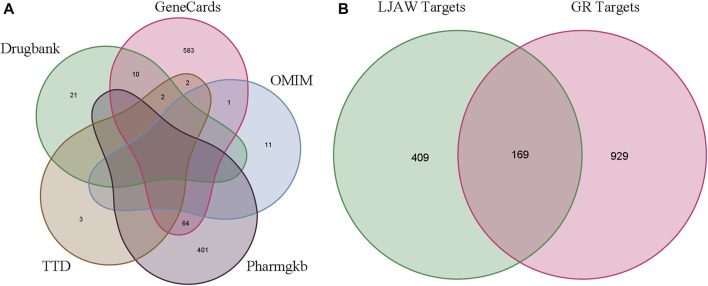
**(A)** Gastrointestinal reactions related targets in five databases. **(B)**Venn diagram of related targets of LJAW and gastrointestinal reactions caused by chemotherapy for colorectal cancer.

A total of 578 human-related gene targets were identified from the Swiss Target Prediction website, and 1,098 disease-related targets were obtained from Drugbank, GeneCards, OMIM, Pharmgkb, and TTD databases (Figure 4A). A Venn diagram was used to intersect the 578 targets predicted by LJAW active ingredients, and 169 common targets were obtained. They were considered potential key targets for LJAW acting on GR caused by chemotherapy for CRC (Figure 4B).

### 3.5 “Ingredient-target” network analysis

**FIGURE 5:**
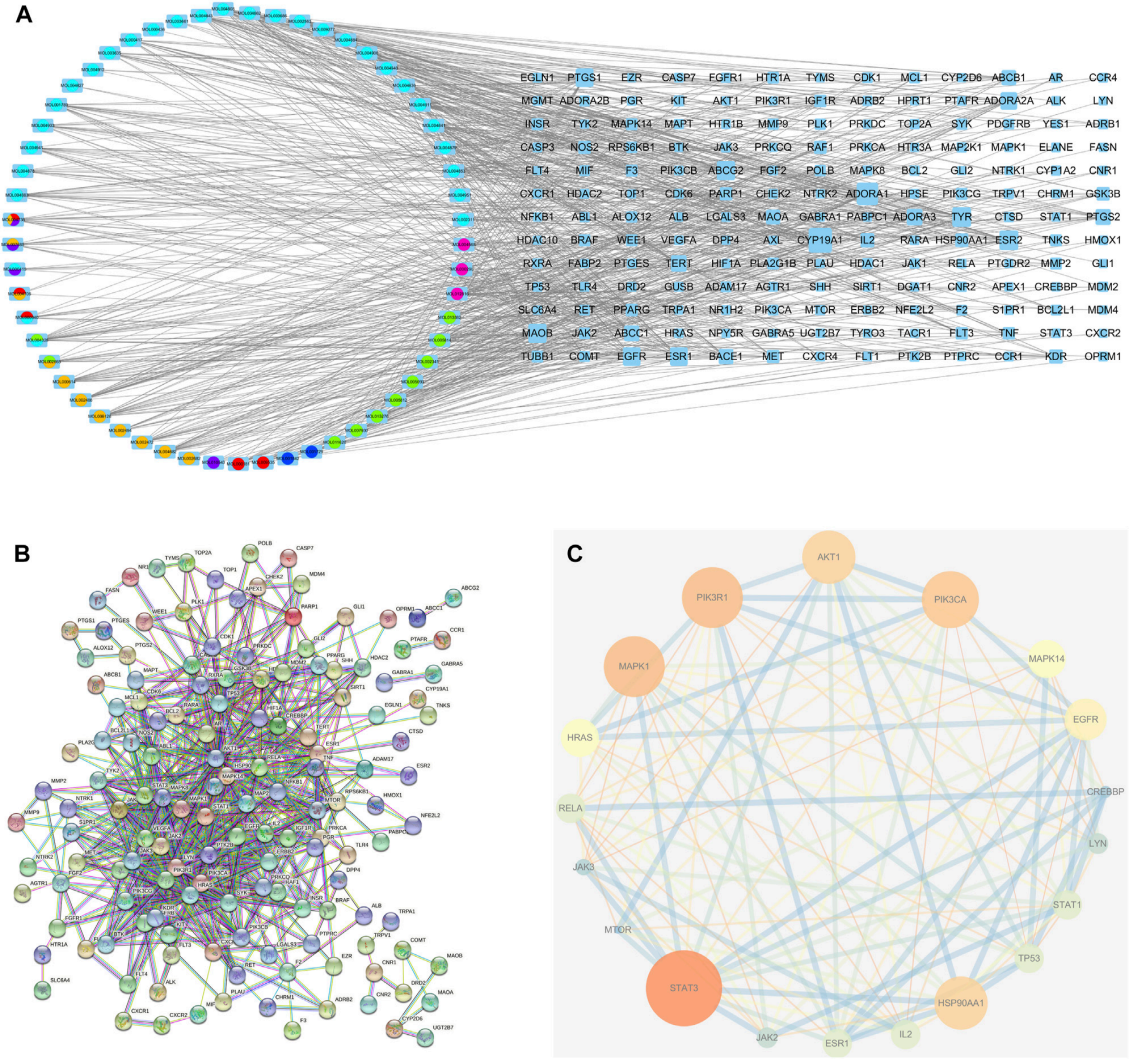
**(A)** “Ingredient-target” interaction network **(B)** Protein-protein interaction network of LJAW in the treatment of gastrointestinal reactions caused by chemotherapy for colorectal cancer. **(C)** Selected 18 core targets.

In total, 97 compounds were screened, but only 57 met the screening conditions. A total of 57 active ingredients and 169 common targets were imported into CytocSape 3.7.2 software to construct the “ingredient-target” network (Figure 5A) made of 266 nodes and 730 edges. The network analysis results showed that the average value of D was 13.78, BC was 1,434.66, and CC was 0.34. There were 18 compounds with values higher than the average ones (Table 2), suggesting that they might be the core active ingredients of LJAW in the treatment of GR caused by chemotherapy for CRC.

**TABLE 2:** Basic information of the core ingredients of LJAW in the treatment of gastrointestinal reactions caused by chemotherapy for colorectal cancer.

Number	Compound	Degree	Betweenness Centrality	Closeness Centrality
1	Galangin	29	3,661.48	0.39130434
2	Licochalcone B	29	2,970.38	0.38071066
3	Glyasperin B	25	3,703.82	0.38071066
4	Licochalcone D	25	2,345.50	0.37815127
5	Retrochalcone	25	2,242.94	0.38330495
6	Pentamethoxyflavone	24	2,370.13	0.37688443
7	Naringenin	24	1847.11	0.37562606
8	ergosterol-5,8-peroxide	23	4,512.82	0.37688443
9	GancaoninE	20	1,485.32	0.36585367
10	Hexahydrocurcumin	19	2,555.07	0.35771066
11	Glabrene	18	2,468.44	0.36585367
12	Licoflavonol	18	1,645.60	0.36000000
13	Methyl octanoate	17	2,831.18	0.36585367
14	Licoisoflavone A	17	2,532.00	0.36585367
15	Osthol	16	2,201.49	0.36000000
16	Phenprobamate	15	2,471.85	0.34992224
17	neryl propionate	15	2,267.84	0.35657686
18	Ferulic acid	14	1793.65	0.34992224

### 3.6 PPI network analysis

A total of 144 nodes and 711 lines were included in the PPI network, indicating that the 169 targets produced a total of 711 interactions (Figure 5B). Cytoscape software was used to visualize the key targets STAT3, STAT1, PIK3CA, PIK3R1, MAPK1, AKT1 for LJAW in the treatment of GR caused by chemotherapy for CRC. In the network, larger nodes represent more effective targets (Figure 5C). The target values of D, BC and CC are shown in Table 3.

**TABLE 3:** Basic information of the key targets of LJAW in the treatment of gastrointestinal reactions caused by chemotherapy for CRC.

Number	Target	Degree	Betweenness centrality	Closeness centrality
1	STAT3	16	15.26031746	0.9
2	STAT1	14	12.65555556	0.818181818
3	PIK3CA	13	9.607142857	0.782608696
4	PIK3R1	13	8.5	0.782608696
5	MAPK1	13	8.499206349	0.782608696
6	AKT1	12	7.462698413	0.75
7	HSP90AA1	12	7.286507937	0.75
8	IL2	12	6.279365079	0.75
9	EGFR	12	5.85	0.75
10	TP53	11	8.391269841	0.72
11	RELA	11	7.417460317	0.72
12	MAPK14	11	6.30952381	0.72
13	ESR1	11	5.66031746	0.72
14	JAK3	10	3.483333333	0.692307692
15	HRAS	10	4.06984127	0.692307692
16	JAK2	10	3.392857143	0.692307692
17	LYN	9	2.927777778	0.666666667
18	MTOR	9	2.08015873	0.666666667
19	CREBBP	7	0.866666667	0.620689655

### 3.7 GO and KEGG enrichment analysis

**FIGURE 6:**
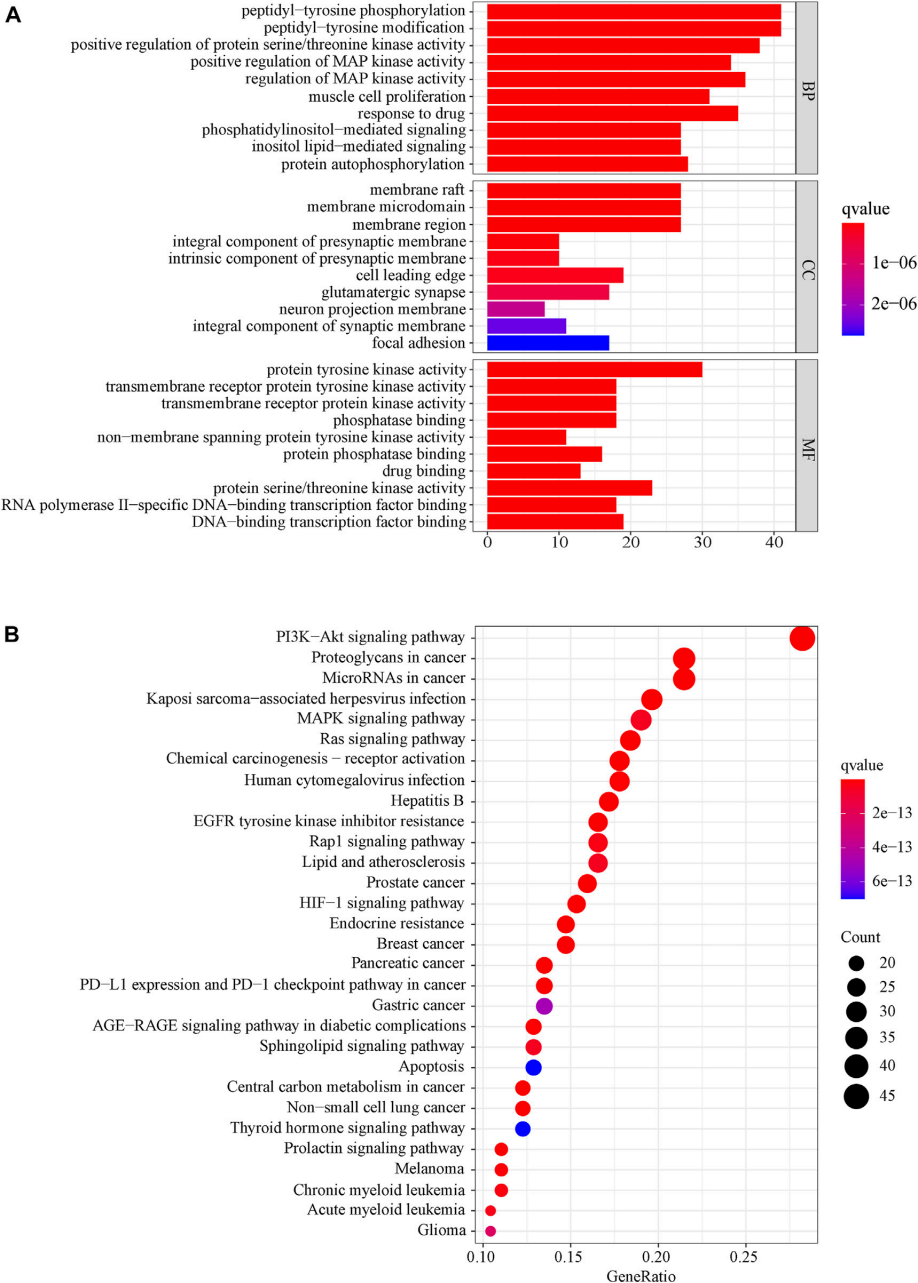
**(A)** Secondary classification histogram of GO functional enrichment of the common targets from LJAW for the treatment of gastrointestinal reactions caused by chemotherapy for colorectal cancer. **(B)** Bubble diagram of KEGG enrichment of the common targets from LJAW for the treatment of gastrointestinal reactions caused by chemotherapy for colorectal cancer. The larger the number of enriched targets, the larger the dots.

We used the DAVID database to enrich the GO bioprocesses of all the 169 common targets. The results of GO enrichment analysis showed that the top BP contains peptidyl−tyrosine, phosphorylation peptidyl-tyrosine modification, and regulation of MAP kinase activity, the top MF contains protein tyrosine kinase activity, protein serine/threonine kinase activity, and DNA−binding transcription factor binding, and the top CC contains membrane raft, membrane microdomain, and membrane region. The top 10 related entries for BP, MF, and CC were shown in a secondary classification histogram using the cluster profiler R package (Figure 6A).

The KEGG pathway enrichment function of the pathways modules in the David database was used to explore the function of 169 common gene targets in signaling pathways involved in the treatment of GR. The top 30 pathways are visually represented in a bubble plot, as shown in Figure 6B. Furthermore, KEGG enrichment results indicated that LJAW might exert therapeutic effects by participating in the regulation of PI3K−Akt signaling pathway, proteoglycans in cancer, MicroRNAs in cancer, Kaposi sarcoma−associated herpesvirus infection, MAPK signaling pathway, and other different pathways.

### 3.8 Molecular docking results

**FIGURE 7:**
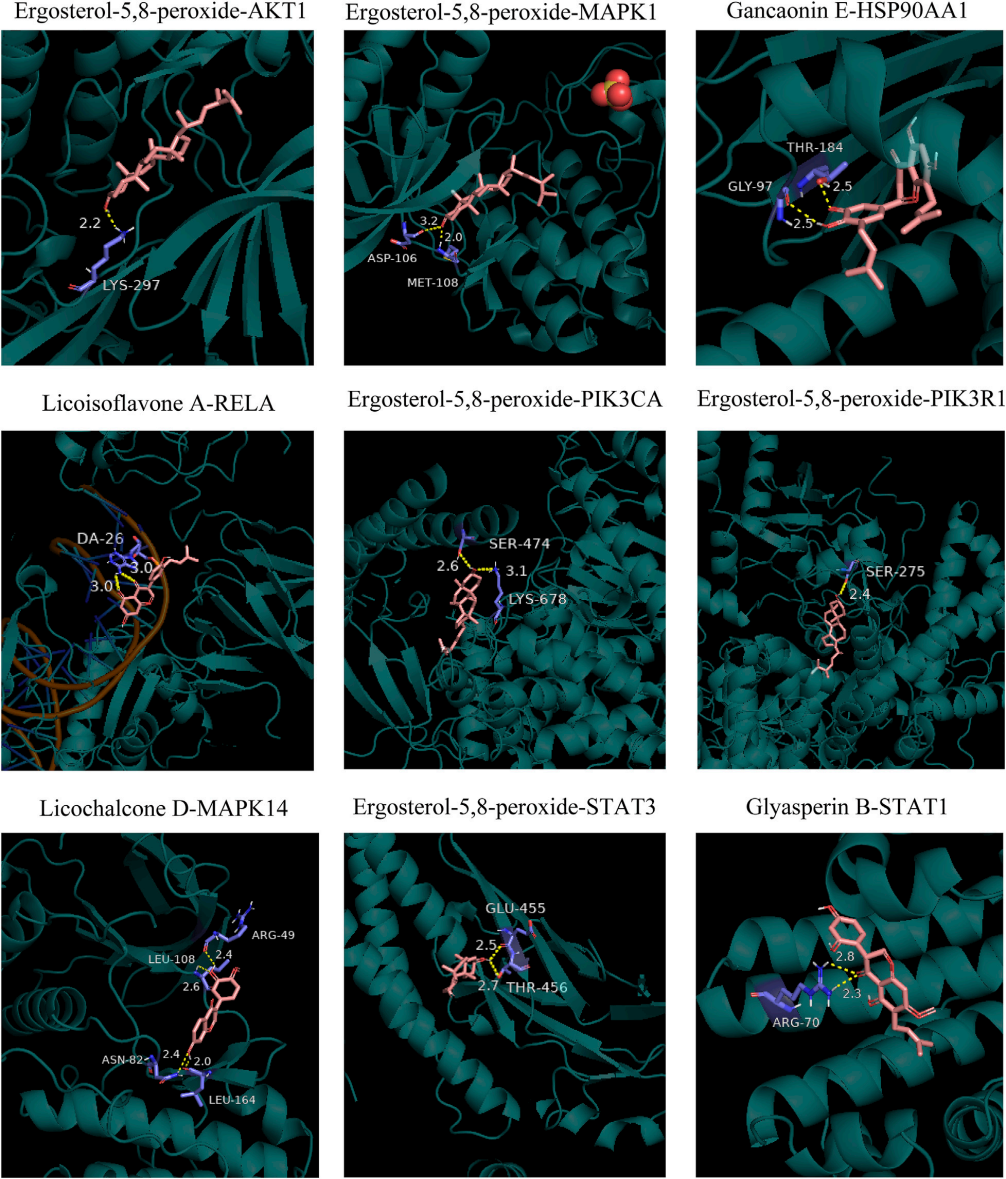
Schematic diagram of docking results between the core ingredients and the key targets.

Molecular docking was conducted between the first 10 core ingredients and the first 12 key targets. Usually, the ingredients and targets have a good binding activity if the binding energy is lower than -5.0 kcal·mol-1. If the binding energy is lower than -7.0 kcal·mol-1, the ingredients and targets have a strong binding activity (Hsin et al., 2013). The molecular docking results showed that the core active ingredients of LJAW and related targets had good binding activity, which confirmed the ingredient-target prediction results. Most of the docking targets with strong binding activity were distributed in PI3K/AKT and MAPK signaling pathways, as shown in Table 4. Some of the docking conformations was are represented in Figure 7.

**TABLE 4:** Molecular docking results.

Number	Molecule name	CAS	Target name	Docking score (kcal/mol)
1	Ergosterol-5,8-peroxide	2061-64-5	AKT1	-11.1
2	Ergosterol-5,8-peroxide	2061-64-5	MAPK1	-10.0
3	GancaoninE	124596-89-0	HSP90AA1	-9.9
4	Licoisoflavone A	66056-19-7	RELA	-9.8
5	Ergosterol-5,8-peroxide	2061-64-5	PIK3CA	-9.7
6	Ergosterol-5,8-peroxide	2061-64-5	PIK3R1	-9.5
7	Licochalcone D	144506-15-0	MAPK14	-8.4
8	Ergosterol-5,8-peroxide	2061-64-5	STAT3	-7.6
9	Glyasperin B	142488-54-8	STAT1	-7.1

### 3.9 Effects of LJAW on the key protein expressions in small intestine and organoids

**FIGURE 8:**
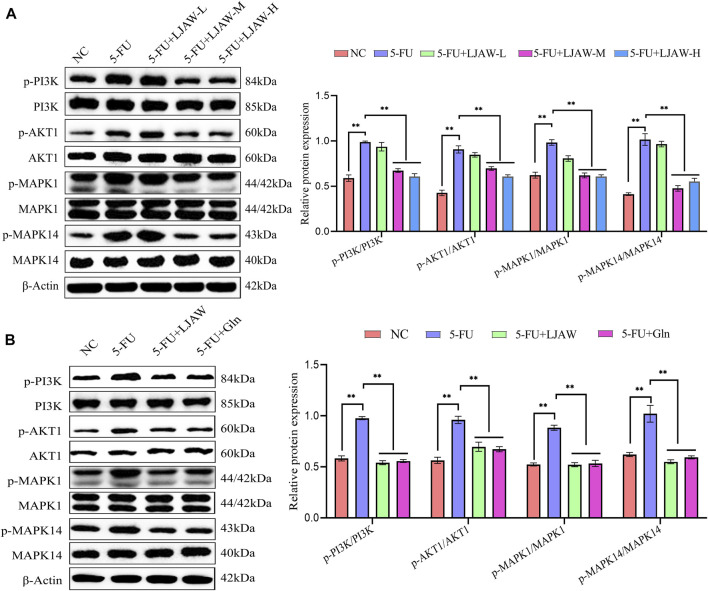
**(A)** Effect of LJAW on the ratios of p-PI3K/PI3K, p-AKT1/AKT1, p-MAPK1/MAPK1, and p-MAPK14/MAPK14 in small intestine **(B)** Effect of LJAW on the ratios of p-PI3K/PI3K, p-AKT1/AKT, p-MAPK1/MAPK, and p-MAPK14/MAPK14 in organoids. Data are expressed as means ± SD (n = 3). **p < 0.01 compared with the 5-FU group.

The expressions of key genes PI3K, AKT1, MAPK1 and MAPK14 and their phosphorylated proteins in PI3K/AKT and MAPK signaling pathway were detected by western bolt to verify the reliability of network pharmacology prediction results. The western bolt results showed that the protein expressions of PI3K, AKT1, MAPK1, and MAPK14 in small intestine and organoids had no significant differences between groups. Compared with the NC group, the 5-FU group had p-PI3K/PI3K, p-AKT1/AKT1, p-MAPK1/MAPK1, and p-MAPK14/MAPK14 ratios in small intestine significantly increased. Differently, the LJAW-M group and the LJAW-H group had p-PI3K/PI3K, p-AKT1/AKT1, p-MAPK1/MAPK1 and p-MAPK14/MAPK14 ratios significantly decreased (Figure 8A). Compared with the NC group, the 5-FU group had p-PI3K/PI3K, p-AKT1/AKT1, p-MAPK1/MAPK1, and p-MAPK14/MAPK14 ratios in organoids significantly increased. Compared with the 5-FU group, the LJAW group had p-PI3K/PI3K, p-AKT1/AKT1, p-MAPK/MAPK1 and p-MAPK14/MAPK14 ratios significantly decreased (Figure 8B).

### 3.10 Effects of LJAW on PUMA in small intestine and organoids

**FIGURE 9:**
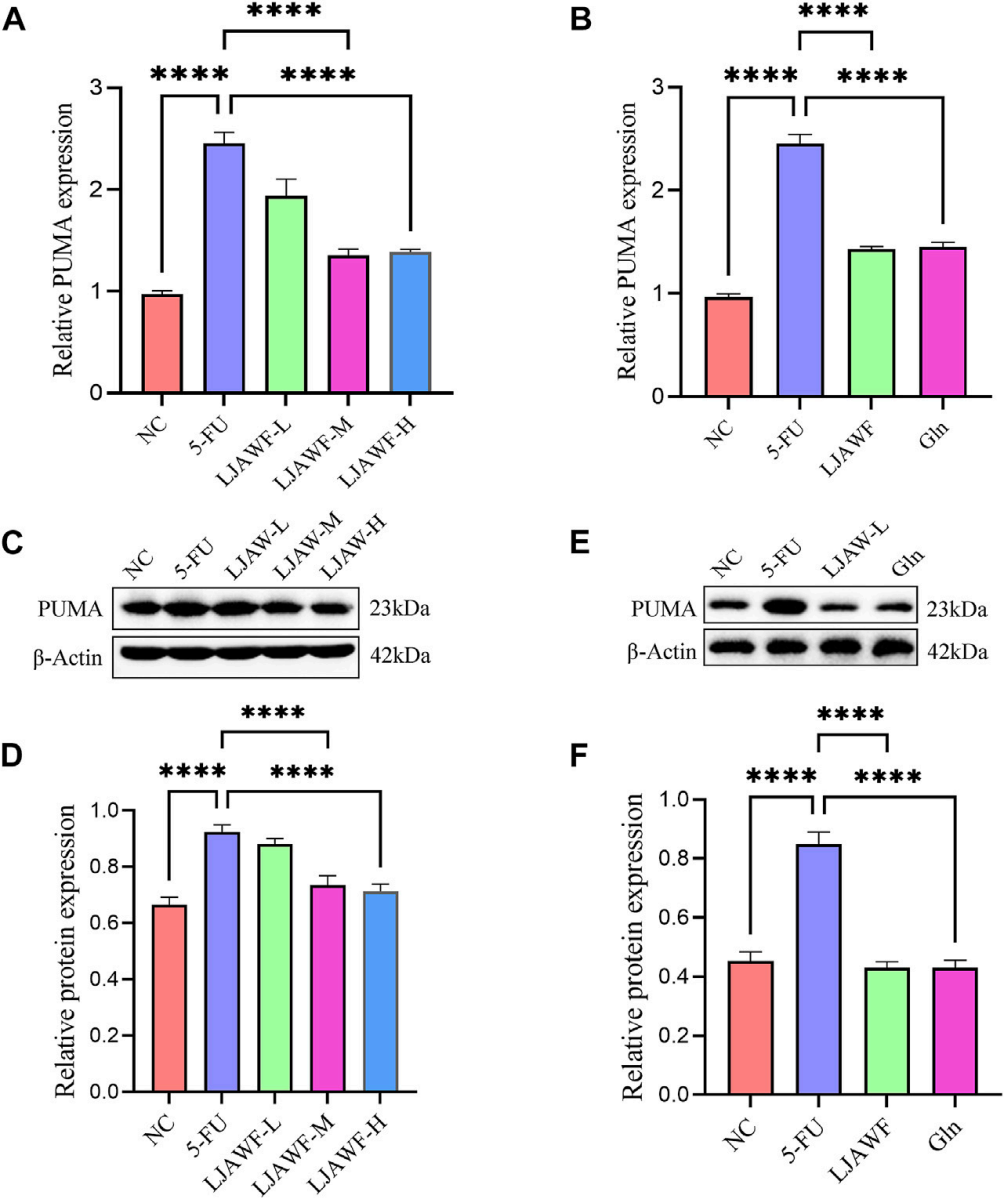
**(A)** and **(B)** The mRNA expression of PUMA in small intestine and organoids after treatment with LJAW **(A)** In small intestine **(B)** In organoids **(C)**–**(F)** The protein expression of PUMA in small intestine and organoids after treatment with LJAW **(C)** and **(D)** In small intestine **(E)** and **(F)** In organoids. Data are expressed as means ± SD (n = 3). ****p < 0.0001 compared with the 5-FU group.

The qRT-PCR results showed that the relative expression level of mRNA for PUMA in small intestine in the 5-FU group was significantly higher than that in the NC group. In the LJAW-M and LJAW-H groups, the expression level was significantly lower than that in the 5-FU group (Figure 9A). Compared with the NC group, the 5-FU group had the relative expression level of mRNA for PUMA in organoids significantly increased. Compared with the 5-FU group, the opposite is valid, and the LJAW group had a relative expression level of mRNA for PUMA significantly decreased (Figure 9B).

The Western Blot results showed that the relative expression of apoptosis-promoting gene PUMA in small intestine in the 5-FU group was significantly increased compared with that in the NC group. In the LJAW-M and LJAW-H groups, the expression was significantly decreased compared with that in the 5-FU group (Figures 9C,D). In comparison with the NC group, the 5-FU group had the relative expression of PUMA in organoids significantly increased, and the LJAW group had the relative expression of PUMA significantly decreased (Figures 9E,F).

## 4 Discussion

Common chemotherapy regimens for CRC include FOLFOX, CapeOX, FOLFIRI that contain anti-tumor 5-FU or drug metabolites with anti-tumor 5-FU precursors. 5-FU often causes intestinal mucosal injury, consequently resulting in gastrointestinal side reactions such as nausea, vomiting, and diarrhea. LJAW has been used alone or combined with other drugs for the treatment of GR caused by chemotherapy for CRC clinically. Our team conducted a retrospective analysis on the cases with gastrointestinal tumors treated with LJAW for the treatment of GR during chemotherapy between March 2003 and February 2016. We found that the effective rate of LJAW in alleviating the adverse GR caused by chemotherapy was 87.5% (Yan et al., 2020).

In this study, UPLC-Q-TOF-MS technology was used for relevant analyses, and as a result, 97 active ingredients in LJAW-IAL were identified. An “ingredient-target” network was constructed with 57 active ingredients and 169 common targets. The results showed that 18 compounds, including galangin, licochalcone B, ergosterol-5, and 8-peroxide, might be the core ingredients of LJAW for the treatment of GR caused by chemotherapy for CRC. Galangin, can increase the activities of antioxidant enzymes SOD, GSH, GPX, and CAT, lower MDA levels, reduce the levels of inflammatory factors TNF-α, IL-1β, and IL-6 and the expression levels of caspase-3 and Bax apoptosis-promoting genes, and enhance the expression of Bcl-2 anti-apoptotic protein by inhibiting MAPK14, JNK, ERK, and NF-κB signaling pathways, thus exerting its solid pharmacological activities, e.g., resisting inflammation and oxidation and reducing apoptosis (Huang et al., 2017; Tomar et al., 2017).

Licochalcone B may decrease LPS-induced NO, TNF-α, and MCP-1 by inhibiting NF-κB signaling pathways and reduce NO, IL-6, and PGE-2 produced by endotoxin-induced macrophages as an anti-inflammatory agent (Furusawa et al., 2009; Fu et al., 2013). Licochalcone B has a strong inhibitory effect on lipid peroxidation and inhibit the ROS production in RAW 264.7 cells induced by lipopolysaccharide (Furusawa et al., 2009).

From a pharmacological perspective, ergosterol-5,8-peroxide has significant anti-inflammatory (Merdivan and Lindequist, 2017). Earlier work has shown that ergosterol-5,8-peroxide decreased the levels of inflammatory factors TNF-α and IL-1α/β in RAW264.7 cells treated with LPS and endotoxin by hampering the activation of MAPK14, JNK, and ERK (Kobori et al., 2007). It is noteworthy that thatergosterol-5,8-peroxide has a strong binding activity to core targets PIK3CA, PIK3R1, AKT1, MAPK1, and MAPK14, with a value of binding energy ranked first. At the same time, the above targets are key targets of KEGG enriched PI3K/AKT and MAPK pathways.

KEGG pathway enrichment results showed that LJAW may attenuate GR caused by chemotherapy for CRC through regulating signaling pathways such as PI3K/AKT, MAPK, and apoptosis. These pathways have been proved to be closely related to the occurrence and development of intestinal injury caused by chemotherapy. Chemotherapy agents such as 5-FU may damage intestinal mucosa by increasing intestinal permeability, promoting the release of inflammatory cytokines, strengthening the oxidative stress response, and accelerating the intestinal epithelial cell apoptosis, thus causing GR (de Barros et al., 2018; Zhang et al., 2018; Yoneda et al., 2021). Li et al. (Zeng et al., 2020) reported that 5-FU might significantly increase the expression levels of apoptosis-related genes such as PUMA and Bax. The significantly advanced expression level of p-AKT protein also indicates that the apoptosis-promoting gene in intestinal cells is activated after chemotherapy. They also pointed out that 5-FU-induced intestinal cell apoptosis is related to the overactivation of PI3K/AKT signaling pathway.

MAPK14 pathway, involved in the regulation of apoptosis in human tissues and mouse intestinal tissues, may induce the activation of related apoptotic genes such as caspase-2, caspase-8, and caspase-3 (Mishra and Karande, 2014; Sheller-Miller et al., 2018). It has been previously confirmed by other related studies that the MAPK14 pathway plays an important role in the pathogenesis of 5-FU-induced intestinal mucosal injury in mice. Scholars have also observed that 5-FU might promote the expression of P-MAPK14 protein, thus, through stimulating the formation and further accumulation of P-p53 protein, 5-FU may promote the expressions of Fas and Bax, and reduce the expression of Bcl-2, this dynamic can boost the activity of the caspase8 promoter, triggering the caspase cascade, including caspase-3, which may ultimately lead to apoptosis of intestinal epithelial cells (Wang et al., 2018; Kordes and Gerling, 2019). Xiang et al. (Xiang et al., 2020) verified the above conclusion and found that in both *in vivo* and *in vitro* experiments, andrographolide, the main active ingredient of herba Andrographitis, can significantly lower the p-MAPK14/MAPK14 ratio after 5-FU treatment as well as the c-caspase-3/caspase-3 and c-caspase-8/caspase-8 ratios, reduce the expression of Bax and increase the expression of Bcl-2. This suggests that andrographolide can inhibit 5-FU-induced intestinal mucosal cell apoptosis by regulating the MAPK14 pathway. Li et al. (Li et al., 2017) found that 5-FU may significantly increase the expressions of IL-6, TNF-α, and IL-1β in mice’s serum and colon tissues and enhance the protein expressions of p-MAPK1 and p-MAPK14 in colon tissues, a phenomenon caused by 5-FU. This indicates that MAPK1 and MAPK14 may actively regulate the expressions of inflammatory factors in the pathogenesis of 5-FU-induced colon injury, thus alleviating the intestinal injury.

An organoid contains various mature cells of specific tissues or organs, so organoid 3D culture technology is promising in research concerning physio pathologies of the body, building disease models, drug screening, and outlining individualized treatment (Rossi et al., 2018; Garreta et al., 2021). Intestinal organoids include all intestinal epithelial cells except for stroma and immune cells, e.g., intestinal stem cells, Paneth cells, goblet cells, neuroendocrine cells, and intestinal epithelial cells. The positions of these different types of cells are consistent with their distribution in the body, and they maintain their corresponding functions, forming a crypt-villus structure (Sato et al., 2009). After passaging, they own stable phenotypic and genetic features. These characteristics are conducive to the dynamic monitoring and study of their physiological and pathological mechanism for repairing the intestinal mucosal injury as an ideal *in vitro* model for exploring the repair of intestinal mucosal injury (Merker et al., 2016; Nikolaev et al., 2020; Ray, 2020). It is rare to find relevant studies on TCM compound intervention in organoids, especially the study on the 5-FU intervention model of intestinal organoids by TCM compound has not been reported. We have made some successful explorations in intervention methods and other aspects.

As a TCM compound, LJAW cannot be directly added to 3D organoids cultured *in vitro*. In recent years, intestinal absorption liquid of drugs has been widely applied in scientific research experiments and showed considerable advantages as a new method for evaluating the pharmacological activity of TCM *in vitro* (Zhang et al., 2012; Zhang J. et al., 2017). The intestinal absorption liquid can eliminate the influence of unabsorbed ingredients on drug efficacy and also avoid the influence of endogenous substances in serum-containing drugs. The number of ingredients in the intestinal absorption liquid is lower than in the original medicinal materials, and the concentrations of ingredients in the intestinal absorption liquid are higher than in the blood, which makes relevant analyses and the identification of active components easier. Studies have proved that all ingredients in the blood may be detected in the intestinal absorption liquid, which points out the possibility that the liquid can be used to screen drugs, evaluate their quality, and perform *in vitro* pharmacological experiments (Liu et al., 2017). At the same time, with the intestinal absorption liquid, the TCM principles can be better analyzed based on the characteristics of the action of TCM prototype components (Zhang et al., 2018). Thus, the reliability and efficiency of research on the mechanism of action for TCM compounds might be improved. In this study, the model of LJAW-IAL intervention in 5-FU-induced intestinal organoid injury was successfully constructed.

Our results showed that LJAW-IAL can reduce morphological destruction of organoids induced by 5-FU. HE results suggested that LJAW can mitigate the destruction of villus height and crypt depth and reduce the infiltration of inflammatory cells in submucosa caused by 5-FU in small intestine. Thus, the protective effect of LJAW on intestinal injury induced by 5-FU was confirmed *in vivo* and *in vitro*. Furthermore, the Western Blot results showed that the protein expressions of the key genes, including PI3K, AKT1, MAPK1, and MAPK14 in small intestine and organoids had no significant differences between groups. Compared with the 5-FU group, p-PI3K/PI3K, p-AKT1/AKT1, p-MAPK1/MAPK1, and p-MAPK14/MAPK14 ratios in the LJAW-M group and the LJAW-H group in small intestine significantly decreased. Compared with the 5-FU group, p-PI3K/PI3K, p-AKT1/AKT1, p-MAPK/MAPK1 and p-MAPK14/MAPK14 ratios in the LJAW group in organoids significantly decreased. The relative expression level of mRNA and protein for apoptosis-promoting gene PUMA in small intestine in the LJAW-M and LJAW-H groups were significantly lower than that in the 5-FU group. Similarly, in comparison with the 5-FU group, the LJAW group had the relative expression level of mRNA and protein for PUMA in organoids significantly decreased. PI3K, AKT1, MAPK1, and MAPK14 are essential targets of PI3K/AKT and MAPK signaling pathways, respectively. These results indicated that LJAW maybe reduce intestinal cell apoptosis by inhibiting the activation of PI3K/AKT and MAPK signaling induced by 5-FU. Despite the organoid being an ideal model for exploring *in vitro* intestinal diseases, nerve cells, immune cells, and related microorganisms in the intestinal microenvironment are not included. Such limitation blocks the application thereof as an experimental model, and hence further investigations shall be conducted in subsequent research on organoids.

## 5 Conclusion

In this study, LJAW has shown significant protective effect on the 5-FU-induced injury of intestinal mucosal and intestinal organoids. To the best of our knowledge, LJAW-IAL was used to intervene in intestinal organoids for the first time. Based on UPLC-Q-TOF-MS technology, network pharmacology and experimental verification, the potential mechanism of LJAW on GR caused by chemotherapy for CRC has been illustrated systematically. Our results revealed that LJAW maybe reduce intestinal cell apoptosis by regulating PI3K/AKT and MAPK signaling pathways, thus alleviating intestinal injury caused by chemotherapy. The multi-ingredient, multi-target, and multi-pathway features of LJAW in the treatment of GR have been successfully clarified. The study results presented here lay a solid foundation for an in-depth study of LJAW. Our results also provide theoretical support for further development and utilization of LJAW.

## Data Availability

The original contributions presented in the study are included in the article/supplementary materials, further inquiries can be directed to the corresponding authors.

## Abbreviations

LJAW, Liu Jun An Wei formula; LJAW-IAL, Liu Jun An Wei formula intestinal absorption liquid; LJAW-H, LJAW high-dose group; LJAW-M LJAW medium-dose group; LJAW-L, LJAW low-dose group; CRC, colorectal cancer; GR, gastrointestinal reactions; TCM, traditional Chinese medicine; PR, Pseudostellariae Radix; AMR, Atractylodis Macrocephalae Rhizoma; PA, Poria; PRA, Pinelliae Rhizoma Praeparatum Cum Zingibere Et Alumine; CRP, Citri Reticulatae Pericarpium; GRER, Glycyrrhizae Radix Et Rhizoma; 5-FU, 5-fluorouracil; UPLC-Q-TOF-MS, ultra high performance liquid chromatography-quadrupole-time of flight tandem mass spectrometry; PPI, protein-protein interaction; GO, Gene Ontology; KEGG, Kyoto Encyclopedia of Genes and Genomes; PDB, protein data bank; BP, biological processes; MF, molecular functions; CC, cell components; D, Degree; BC, Betweenness Centrality; CC, Closeness Centrality.
